# Dieulafoy lesion of the gallbladder: A rare cause of hemobilia and acute pancreatitis – Case report

**DOI:** 10.1016/j.ijscr.2019.12.004

**Published:** 2019-12-16

**Authors:** Teresa Santos, Marta Serra, António Oliveira, Catarina Fernandes

**Affiliations:** Centro Hospitalar do Baixo Vouga – Av. Artur Ravara, 3810 – 501 Aveiro, Portugal

**Keywords:** GI, gastrointestinal, BRB, bilirubin, AF, alkaline phosphatase, CT, computed tomography, MRI, magnetic resonance image, ERCP, endoscopic retrograde cholangiopancreatography, Dieulafoy lesion, Gallbladder, Hemobilia, Acute pancreatitis, Intraoperative cholangiogram, Case report

## Abstract

**Introduction:**

Dieulafoy lesion of the gallbladder is very rare. It can cause hemobilia and, consequently, acute pancreatitis. After extensive research, the authors found six reported cases of this entity, none of them presenting as acute pancreatitis.

**Case presentation:**

A 78-year-old woman presented to the emergency department a right upper-quadrant pain and nausea. Abdominal ultrasound showed a gallbladder full of a heterogenic content with no signs of lithiasis or acute cholecystitis. Blood lipase was significantly elevated. She was admitted for acute pancreatitis. During hospitalization she developed jaundice and hematemesis requiring blood transfusion. The upper gastrointestinal (GI) endoscopy with side-view duodenoscopy showed blood through the papilla of Vater. Computed tomography and magnetic resonance imaging confirmed the content of the gallbladder with no changes in the biliary tree. A laparoscopic cholecystectomy with intraoperative cholangiogram was performed. Histology revealed a Dieulafoy lesion. The patient was discharged three days after surgery and had no subsequent episodes.

**Conclusion:**

Hemobilia should be considered as a cause of upper GI bleeding and acute pancreatitis, especially if both are concurrent. Treatment is directed to the cause, with bleeding control and restoration of bile flow, which can be accomplished by a single minimally invasive surgery.

## Highlights

• Dieulafoy lesion of the gallbladder is a very rare cause of hemobilia.
• Hemobilia should be considered as a cause of acute pancreatitis.
• Intraoperative cholangiogram is effective in flushing small biliary clots.

## 1 Introduction

Hemobilia is a rare cause of gastrointestinal bleeding and can arise from any part of the biliary tree, gallbladder, pancreas or ampullary orifice. The most common cause is iatrogenic injury followed by hepatobiliary tumors [1].

The typical clinical features of abdominal pain, jaundice, and gastrointestinal bleeding, known as the “Quinke triad” [2], is present in 22–38% of cases [3].

Hemobilia usually involves minor bleeding and stops spontaneously. Sometimes, the bleeding is more intense and the blood flows into the duodenum, presenting as melena or hematemesis [4].

A Dieulafoy lesion is characterized by a vessel with an abnormally large caliber that runs a tortuous course beneath the mucosa. A defect within this layer allows the vessel to protrude and thus, to bleed. It can be found anywhere throughout the gastrointestinal tract, but it is very rarely found in the gallbladder, with only six reported cases. This entity presents as acute hemorrhage, sometimes recurrent [5].

Blood in the biliary tree tends to form clots [4] because differences in specific gravity and surface tension between blood and bile prevent the two from mixing. Clot formation may consequently result in biliary obstruction or pancreatitis [6].

Diagnosis requires a high index of suspicion and exams to be requested depend on clinical presentation.

Treatment directed to the cause, bleeding control and restoration of bile flow are the therapeutic mainstays.

We present a case of acute pancreatitis due to a Dieulafoy lesion of the gallbladder. This work is reported in line with the SCARE criteria [7].

## 2 Case presentation

A 78-year-old diabetic, hypertensive woman, with stage IV chronic kidney disease and chronic anemia [basal hemoglobin (Hb) level: 9 g/dL)] presented to the emergency department a one-week history of right upper quadrant and epigastric pain associated with vomit. She had no previous similar complains or history of biliary lithiasis. She had no alcoholic habits and was not taking any new medicine. She had no trauma or biliary tract manipulation history. No fever, jaundice or stool alterations were reported.

Abdominal palpation showed epigastric pain but no tenderness or peritoneal signs.

Blood count revealed anemia (Hb = 9,1 g/dL) and normal leucocytes. Biochemistry showed normal bilirubin (BRB) and augmented transaminase (AST: 714U/L; ALT: 405U/L) and alkaline phosphatase (AF): 660U/L. Lipase was 1659 U/L (N: 53 U/L).

**Fig. 1:**
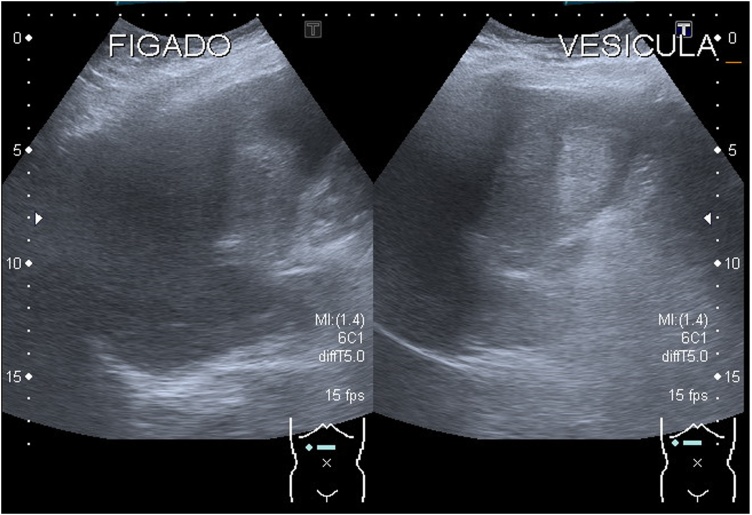
Abdominal Ultrasound image showing a heterogenic content of the gallbladder, with no acoustic shadow. “FIGADO” means liver. “VESICULA” means gallbladder.

Abdominal ultrasound showed a gallbladder full of a heterogenic hyperechoic content approximately 8,5 cm long, with no acoustic shadow and a normal biliary tree (Fig. 1).

The patient was admitted with the diagnosis of acute pancreatitis based on pain and abnormal lipase values.

During the first days the patient had a favourable clinical outcome. One week after admission, whenever dietary progression was attempted, there was relapsing epigastric pain, once associated with severe asthenia and overt hematemesis requiring nasogastric intubation. Vital signs were normal and nasogastric drainage was self-limited, of about 200 millilitres. Hemoglobin level dropped to 6,5 g/dL and there was an increase in AF to 804 U/L and BRB level (Total BRB: 5,72 mg/dL; Direct BRB: 4,69 mg/dL). Two units of packed red blood cells were transfused, and an upper GI endoscopy was requested. This exam showed residual blood in stomach and duodenum but no mucosal lesions. A side-view duodenoscopy was performed, and dark blood was seen through the papilla of Vater.

To study possible causes of hemobilia, an abdominal computed tomography (CT) (with no contrast duo to renal impairment) and magnetic resonance cholangiography and abdominal MRI were requested.

**Fig. 2:**
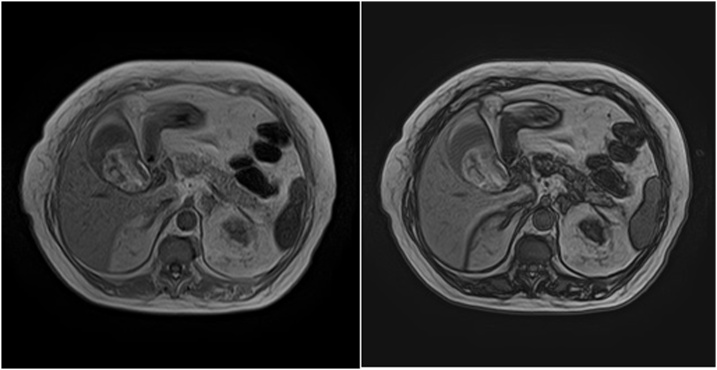
T1 MRI images showing a heterogenic in-phase and out-of-phase hyperintense content of the gallbladder and a thin and homogeneous gallbladder wall.

**Fig. 3:**
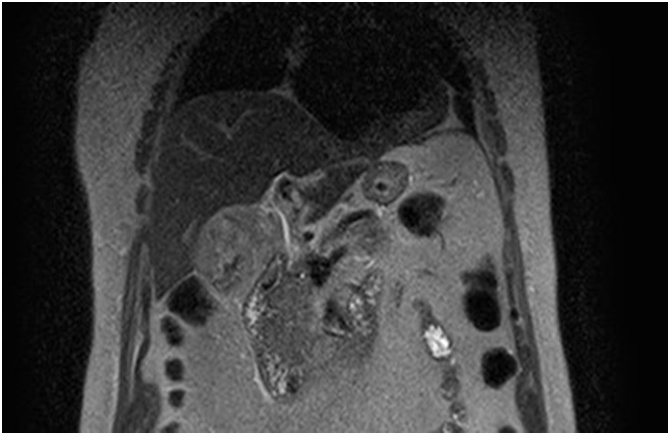
Magnetic resonance cholangiography showing a normal biliary tree.

The first showed a heterogenic content of the gallbladder and no alterations in the biliary tree. The second (one week after hematemesis episode) confirmed the content and showed a gallbladder wall homogeneously thin (Fig. 2). The biliary tree had normal diameter and no signs of lithiasis (Fig. 3).

Meanwhile, although overt hematemesis passed, epigastric complaints and anemia persisted, requiring transfusion of a total of six packed red cells.

**Fig. 4:**
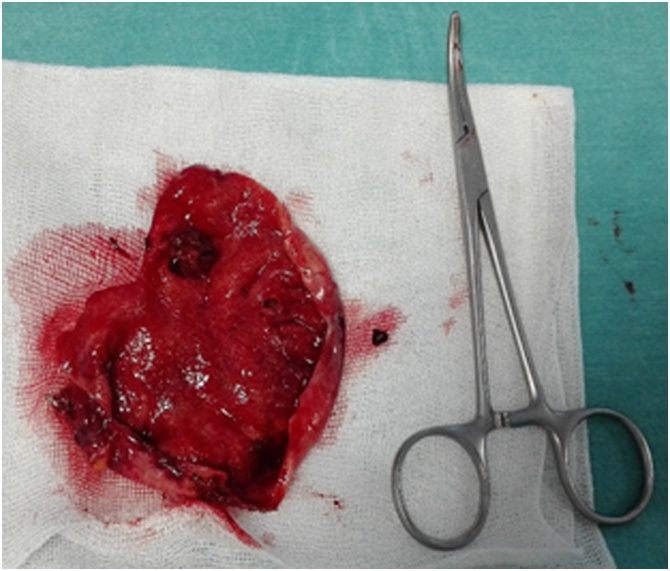
Open gallbladder with no gallstones but blood clots and an extra mucosal hemorragic lesion.

The gallbladder, which had no signs of malignancy, was considered the source of hemobilia. Nineteen days after admission, once the patient recovered from acute anemia and abdominal discomfort, a laparoscopic cholecystectomy with intraoperative cholangiogram was performed. No alterations were seen in the biliary tree. The gallbladder was full of blood clots and a hemorrhagic lesion was seen (Fig. 4).

The patient was discharged three days after surgery, asymptomatic, and with improved cholestatic markers (AF: 488 U/L, Total BRB: 1,36 mg/dL; Direct BRB 1,16 mg/dL).

**Fig. 5:**
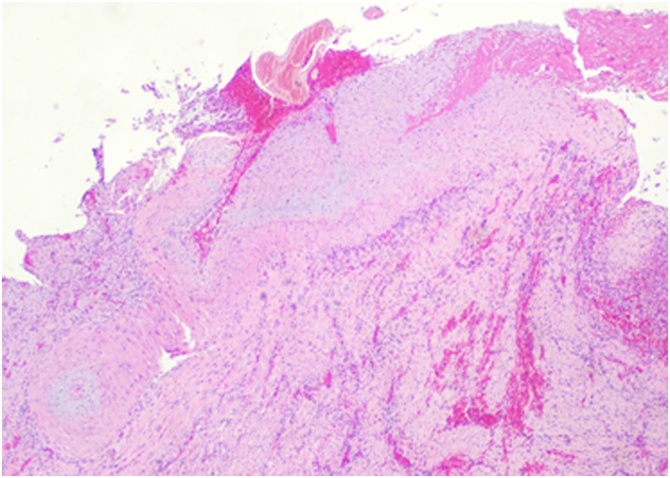
HE 100× showing a tortuous, large caliber muscular artery beneath the mucosa, which is ulcerated.

Histology revealed a Dieulafoy lesion of the gallbladder (Fig. 5).

A follow-up of one and a half year was considered and no subsequent biliary episodes have occurred. The patient has now normal citocholestatic markers and no ultrasonographic changes in the biliary tree.

## 3 Discussion

In this case, hematemesis in a patient with acute pancreatitis of undetermined cause, along with elevation of cholestatic markers and an unremarkable upper GI endoscopy, raised suspicion of hemobilia and lead to a side-view duodenoscopy which confirmed the diagnosis.

As other common causes of acute pancreatitis were excluded, hemobilia was assumed as its cause. In fact, after treatment, cholestatic markers improved and the patient has never had other episodes.

Hemobilia diagnosis requires a high index of suspicion. Its treatment is directed to the cause and bile flow must be warranted.

Iatrogenic cause and trauma were excluded during clinic interview. Evaluation of liver, gallbladder, pancreas and biliary tree was then mandatory.

Abdominal ultrasound, computed tomography, magnetic resonance imaging, angiography and endoscopic retrograde cholangiopancreatogram (ERCP) are available means for etiologic study.

Ultrasound has low sensitivity and specificity for hemobilia but, in this case, it was important to favour the hypothesis of blood as the content of the gallbladder. Clots in the gallbladder are usually hyperechogenic without acoustic shadow and do not change with position [8].

CT is sensitive to detect abnormalities in the biliary tree, gallbladder and pancreas, as well as active bleeding, especially if contrast enhanced, which was not the case in this patient due to renal impairment. MRI, in addition, is especially sensitive to detect bile duct dilatation or distortion and can also distinguish blood from stones and sludge [9].

ERCP is a valuable exam as it allows direct visualization and biopsies. Following sphincterotomy and cholangiogram, blood clots can be removed with balloon catheters or stone retrieval baskets. Drainage can be left for monitoring haemorrhage, irrigation of bile duct or just for decompression or prevention of further infection [1].

Angiography is an effective diagnostic and therapeutic tool for hemobilia, especially if there is massive bleeding. However, it is not always available, and neither is embolization always successful [10]. It is normally needed if the diagnosis is in doubt or other investigations are not contributory.

Most cases of hemobilia cease spontaneously due to its intermittent nature and require volume replacement or blood transfusion only. However, when the source of the hemorrhage is a Dieulafoy lesion, sometimes an emergent cholecystectomy may be necessary, as it was in four out of the six published cases [5].

Biliary flow may be warranted by removing or flushing blood clots, which is most frequently accomplished by ERCP.

In this case, gallbladder was the source of the hemorrhage and, as cancer was not suspected, a laparoscopic cholecystectomy was performed. Intraoperative cholangiogram allowed for biliary tree re-evaluation and flushing of any millimetric blood clots. As post-operative cholestatic markers improved, ERCP was not necessary, saving the patient the need of another invasive procedure that would have compromised the sphincter of Oddi.

The prognosis of hemobilia is largely dependent on the underlying causes. In this case, a laparoscopic cholecystectomy with intraoperative cholangiogram was curative. There is no need for long-term follow-up. Despite this, we re-evaluated the patient one and a half year after admission and both the cholestatic markers and the biliary tree diameter were within normal range.

## 4 Conclusion

Dieulafoy lesion of the gallbladder is very rare and can be considered as a cause of upper GI bleeding and acute pancreatitis, especially if both are concurrent. It can be treated by laparoscopic cholecystectomy with intraoperative cholangiogram. This treatment is effective and possible as a single minimally invasive surgery, with no need for further invasive procedures.

## Sources of funding

Nothing to declare.

## Ethical approval

In my institution (Centro Hospitalar do Baixo Vouga, Aveiro, Portugal) the publication of clinical cases, especially those that do not expose the identity of the patient, does not require ethical approval.

## Consent

Written informed consent was obtained from the patient for publication of this case report and accompanying images.

## Author contribution

Teresa Santos, first and corresponding author, was responsible for conceptualization, methodology, writing – Original Draft, Writing – Review & Editing

Marta Serra contributed to data research.

António Oliveira and Catarina Fernandes were the surgeons responsible for the patient.

All authors read and approved the final manuscript.

## Registration of research studies

Not applicable – case report.

## Guarantor

Teresa Santos

## Provenance and peer review

Editorially reviewed, not externally peer-reviewed.

## Declaration of Competing Interest

The authors have no financial, consultative, institutional or other relationships that might lead to bias or conflict of interest.
